# Effect of Electronic Outreach Using Patient Portal Messages on Well Child Care Visit Completion: A Randomized Clinical Trial

**DOI:** 10.1001/jamanetworkopen.2022.42853

**Published:** 2022-11-18

**Authors:** Anne E. Berset, Mary Carol Burkhardt, Yingying Xu, Anne Mescher, William B. Brinkman

**Affiliations:** 1 Division of General & Community Pediatrics, Cincinnati Children’s Hospital Medical Center, Cincinnati, Ohio; 2 Department of Pediatrics, University of Cincinnati College of Medicine, Cincinnati, Ohio

## Abstract

**Importance:**

Outreach messages to patients overdue for well child care (WCC) can be delivered different ways (ie, telephone calls and text messages). Use of electronic health record patient portals is increasingly common but their effectiveness is uncertain.

**Objective:**

To determine the effectiveness of patient portal outreach messages, with and without the date of the last WCC, on the scheduling and completion of WCC visits and completion of vaccinations.

**Design, Setting, and Participants:**

An intention-to-treat, multigroup, randomized clinical trial was conducted at 3 academic primary care practices from July 30 to October 4, 2021. The population included predominantly non-Hispanic Black, low-income children (age, 6-17 years) whose parent had an active portal account.

**Interventions:**

Participants were randomized to the standard message, tailored message, or no message (control) group. Two messages were delivered to those in the message groups.

**Main Outcomes and Measures:**

Outcomes included WCC visit scheduled within 2 weeks of the first intervention message, WCC visit completed within 8 weeks (primary outcome), and receipt of COVID-19 vaccine within 8 weeks.

**Results:**

Nine hundred forty-five patients participated (mean [SD] age, 9.9 [3.3] years, 493 [52.2%] girls, 590 [62.4%] non-Hispanic Black, 807 [85.4%] publicly insured). Scheduling rates were 18.4% in the standard message group (adjusted risk ratio [aRR], 1.97; 95% CI, 1.32-2.84) and 14.9% in the tailored message group (aRR, 1.57; 95% CI, 1.02-2.34) compared with the control group (9.5%). Well child care visit completion rates were 24.1% in the standard message group (aRR, 1.92; 95% CI, 1.38-2.60) and 19.4% in the tailored message group (aRR, 1.52; 95% CI, 1.06-2.13) compared with the control group (12.7%). Among eligible children, rates of receiving the COVID-19 vaccine were 16.7% in the standard message group compared with 4.8% in the tailored message (aRR, 3.41; 95% CI, 1.14-9.58) and 3.7% in the control groups (aRR, 4.84; 95% CI, 1.44-15.12).

**Conclusions and Relevance:**

In this randomized clinical trial, outreach messages delivered via electronic health record patient portals increased the rates of scheduling and completing WCC visits and receiving the COVID-19 vaccine, providing a useful tool to help restore WCC in populations whose care was delayed during the pandemic.

**Trial Registration:**

ClinicalTrials.gov Identifier: NCT04994691

## Key Points

**Question:**

Does reminder messaging delivered via electronic health record patient portal affect the scheduling and completion of well child care (WCC) visits and delivery of vaccinations?

**Findings:**

In this randomized clinical trial that included 945 patients aged 6 to 17 years, the rates of scheduling and completing a WCC differed significantly between groups, with the portal message groups outperforming the control group. Rates of COVID-19 vaccine completion also significantly differed between groups.

**Meaning:**

The findings of this randomized clinical trial show that outreach patient portal messages increased the rates of scheduling and completing WCC visits and receiving some vaccinations.

## Teaser

This randomized clinical trial examines the use of patient portals to aid in scheduling and completion of well child care visits and vaccinations.

## Introduction

Preventive services were delayed during the COVID-19 pandemic, including well child care (WCC).^1,2,3^ Following pandemic shutdowns, vaccination rates decreased,^4,5,6^ with rates for non-Hispanic Black children disproportionately worsening^6^ and vaccine hesitancy increasing.^7,8^ Pervasive mistreatment, systemic racism, and neglect of Black and Latinx communities by the medical system have negatively impacted vaccine uptake among these populations.^9,10^ Outreach approaches may help in bringing children back for care, including text messages, telephone calls, mailings, school campaigns, community navigators, and home visitors.^11^ For example, reminders sent by letter and/or automated telephone calls have been reported to improve WCC visit completion rates among pediatric patients.^12,13,14^

Electronic health record (EHR) portal messaging is a newer method to send outreach messages. Patient portals are secure online applications that give patients and parents 24-hour EHR access. Portal use in pediatric and adult primary care settings has increased since 2008.^15,16,17^ Portal reminders to schedule influenza vaccines among pediatric^18,19^ and adult^20,21,22,23^ patients have been minimally effective. Portal messages have successfully increased the scheduling, but not completion, of wellness visits among adults.^24^ Yet little is known about the effect of patient portal messages on the scheduling and completion of overdue pediatric WCC visits and research has highlighted racial, ethnic, and insurer disparities in portal activation and use among pediatric patients.^25,26^ To our knowledge, no pediatric studies have tested EHR portal messaging to increase WCC visits despite its potential to increase access by enabling direct scheduling of appointments, eliminating the constraint of calling during business hours.

We sought to determine the effect of EHR patient portal outreach messages, with and without the date of the last WCC, on the scheduling and completion of WCC visits among patients aged 6 to 17 years overdue for annual WCC visits. We tailored messages to include the date of the last WCC to address distortions of time perception experienced during the pandemic.^27,28,29^ We hypothesized that outreach messages would be superior to no message, and messages with tailoring would outperform standard messages. Secondary outcomes were vaccination rates for eligible patients.

## Methods

### Study Design and Randomization

We conducted a multigroup, randomized clinical trial from July 30 to October 4, 2021. One of us (Y.X.) generated a random allocation sequence stratified by clinic location using block randomization (with a block size of 3) and randomly assigned eligible patients (1:1:1) to 1 of 3 groups: standard message, tailored message, or control group (no message). The Cincinnati Children's Hospital Medical Center Institutional Review Board approved the study, granting a waiver of informed consent because there was minimal risk and the data were deidentified. We provide the trial protocol and statistical analysis plan in Supplement 1. This study followed the Consolidated Standards of Reporting Trials (CONSORT) reporting guideline.

### Setting

The study occurred at 3 academic pediatric primary care practices. These practices serve a predominantly non-Hispanic Black, low-income population and provide more than 60 000 visits annually to 30 000 patients. The COVID-19 vaccine was available and could be given at WCC, ill, or vaccine-only encounters. At the start of the study, 44.4% of the patients had active portal accounts (ie, registered username and password). At age 13 years, patients gain account access and can sign assent designating at least 1 parent as a proxy, allowing parents to also receive portal messages. Parents can schedule appointments using the portal self-scheduling system available 24 hours daily or by calling the scheduling center Monday through Friday, 8:30 am to 5:00 pm.

### Population

We included patients aged 6 to 17 years who met the following eligibility criteria: (1) seen at 1 of our 3 primary care practices within 2 years, (2) no WCC visit in the past 365 days, (3) no WCC visit scheduled, (4) preferred language of English and/or Spanish, and (5) active EHR portal account. We downloaded an EHR report of potentially eligible patients that was limited to those aged 6 to 17 years who had not had a WCC in the past year. We collected the following EHR variables to characterize our sample and for consideration as covariates in analyses: parent communication preference; time since last WCC; absence of past patient receipt of measles, mumps, and rubella (MMR) vaccine or diphtheria, tetanus, and acellular pertussis (DTaP) vaccine as a proxy for declining childhood vaccinations; patient lifetime historical institutional no-show rate; and practice appointment availability. We also tracked the community incidence of COVID-19.^30^

### Interventions

We sent 2 patient portal messages (ie, between 11:30 am and 2:30 pm on Monday and Thursday) to each of the messages groups in 2 sequential weeks through our institution’s EHR portal (MyChart; Epic Systems Corp), using the parent’s preferred language. Parent preferences are elicited and recorded when first registered in the EHR. Parents receive an email informing them of a message in the patient portal and a link to log in and access the message.

We crafted the messages based on the extant literature^11^ and feedback from parents, as well as outreach medical assistants who call and text with families daily. Examples of patient portal messages are given in eTable 2 in Supplement 2. The standard messages referenced the patient’s first name, reminded parents their child was due for a WCC visit, and asked them to schedule using the portal or by calling the number provided. The tailored messages included the information in the standard message, and additionally included the date of the patient’s last WCC and patient age. We randomly assigned the standard message group to receive the intervention the first week and the tailored message group the following week. Interpreter services translated our messages into Spanish. One of us (A.E.B.) completed a medical record audit at the end of the study to determine whether messages had been read.

### Outcomes

Our primary outcome was WCC visit completion within 8 weeks of the date we sent the first message or the date of randomization for the control group. Secondary outcomes included appointment scheduled within 2 weeks and receipt of first COVID-19 vaccination within 8 weeks among eligible patients (aged ≥12 years at the time of the study). Post hoc secondary outcomes included patient receipt of DTaP vaccine, human papillomavirus (HPV) vaccine, and meningococcal conjugate vaccine (MCV4) among eligible patients. An exploratory analysis examined whether children in the tailored message group who did and did not complete a WCC visit differed on time since the last WCC. We assessed all outcomes using EHR data.

### Sample Size

We calculated the study sample size based on the hypothesis that 20% of patients in the standard message, 30% of patients in the tailored message, and 2% of those in the control group would complete a WCC visit within 8 weeks. We based our hypotheses on effect sizes documented for automated text reminders on immunization rates.^11^ We calculated the sample size based on the hypothesized proportions. To detect a difference in WCC visit completion for all 3 comparisons (standard vs control, tailored vs control, and standard vs tailored) with 80% power at 5% significance level, we required a sample size of 293 per group (879 participants overall). We blinded clinical practice teams and our outcome assessor by concealing group allocation.

### Statistical Analysis

We conducted descriptive analyses to characterize the participants in terms of demographic characteristics and other potential covariates. We conducted intention-to-treat analysis to compare outcomes among the 3 groups. We used logistic regression models, adjusting for characteristics that meaningfully differed across the groups, to examine each of our binary outcomes. We obtained odds ratios and then converted to risk ratio (RRs) using standard methods,^31^ with statistical significance defined as 2-sided *P* < .05. Because some patients were not sent messages as intended, we conducted a per-protocol analysis, excluding patients who were not sent at least 1 message. Because our study design did not account for siblings or others living in the same household, we also conducted sensitivity analyses for our intention-to-treat and per protocol analyses, excluding patients in the control group who shared the same telephone number and/or proxy (parent on the account) with another patient allocated to a message group. We conducted all analyses using SAS, version 9.4 (SAS Institute Inc).

## Results

### Baseline Characteristics

**Figure 1.**
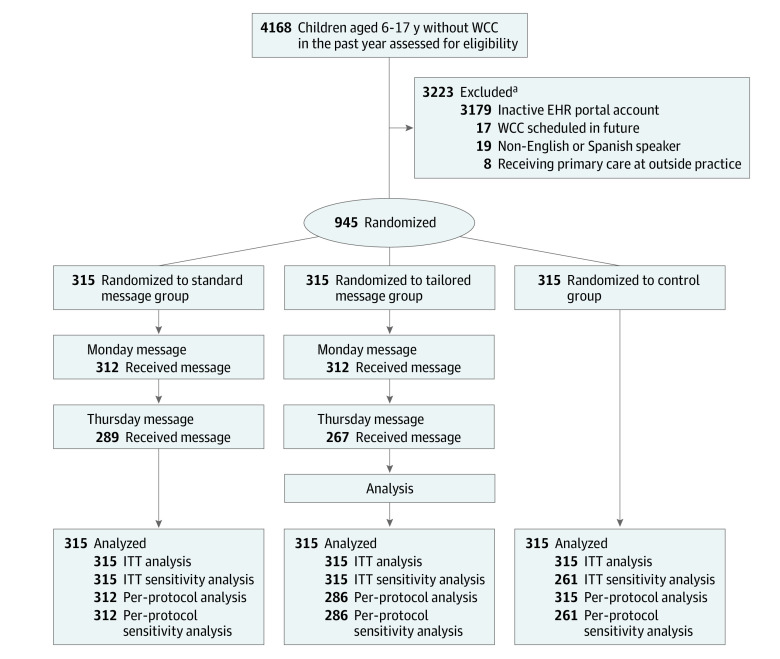
Consolidated Standards of Reporting Trials Diagram EHR indicates electronic health record; ITT, intention-to-treat; and WCC, well child care. ^a^Patients excluded had higher percentages of younger, Black, and self-pay and publicly insured patients compared with enrolled patients.

Of the 945 children (493 [52.2%] girls and 452 [47.8%] boys) who were eligible and randomized (Figure 1), most were non-Hispanic Black (590 [62.4%]) and had public insurance (807 [85.4%]) (Table 1). Mean (SD) age was 9.9 (3.3) years. We observed meaningful differences between the groups on insurance status, which we adjusted for in subsequent analyses. The estimates from adjusted models and those from unadjusted models were very close, so we present adjusted estimates herein and provide unadjusted estimates in eTable 1 in Supplement 2. Few patients (1.0%) appeared to have declined childhood vaccines based on our proxy measure of not having received at least 1 past dose of MMR and DTaP. The mean time since last WCC was 17 months (range, 12-24 months). All practice locations maintained WCC appointment availability less than or equal to 30 days throughout the study (median, 12 days; range 1-30 days). We were unable to accurately ascertain when parents read the patient portal message because the EHR time stamp updated anytime the message was viewed. Thus, we did not pursue further analysis. The Delta variant of SARS-CoV-2 spread in our region during the study time frame, with COVID-19 community 7-day moving average increasing from 94 to 568 per 100 000.

**Table 1.** Baseline Characteristics

Characteristic	No. (%)			
	Standard message	Tailored message	Control group	Total
Unique patients, No.	315	315	315	945
Age range, y				
6-8	133 (42.2)	134 (42.5)	137 (43.5)	404 (42.8)
9-11	98 (31.1)	98 (31.1)	96 (30.5)	292 (30.9)
12-14	40 (12.7)	38 (12.1)	33 (10.5)	111 (11.7)
15-17	44 (14.0)	45 (14.3)	49 (15.6)	138 (14.6)
Sex				
Female	152 (48.3)	167 (53.0)	174 (55.2)	493 (52.2)
Male	163 (51.7)	148 (47.0)	141 (44.8)	452 (47.8)
Race and ethnicity				
American Indian or Alaska Native, non-Hispanic	0	1 (0.3)	0	1 (0.1)
Asian, non-Hispanic	10 (3.2)	4 (1.3)	5 (1.6)	19 (2.0)
Black, non-Hispanic	184 (58.4)	195 (61.9)	211 (67.0)	590 (62.4)
Black, Hispanic	3 (1.0)	1 (0.3)	2 (0.6)	6 (0.6)
Middle Eastern, non-Hispanic	0	1 (0.3)	0	1 (0.1)
Multiracial, non-Hispanic	21 (6.7)	15 (4.8)	15 (4.8)	51 (5.4)
Multiracial, Hispanic	1 (0.3)	1 (0.3)	1 (0.3)	3 (0.3)
Native Hawaiian and other Pacific Islander, Hispanic	0	0	1 (0.3)	1 (0.1)
White, non-Hispanic	82 (26.0)	73 (23.2)	71 (22.5)	226 (23.9)
White, Hispanic	5 (1.6)	10 (3.2)	3 (1.0)	18 (1.9)
Missing data, non-Hispanic	3 (1.0)	3 (1.0)	1 (0.3)	7 (0.7)
Missing data, Hispanic	6 (1.9)	11 (3.5)	5 (1.6)	22 (2.3)
Insurance				
Public (ie, Medicaid)	280 (88.9)	275 (87.3)	252 (80.0)	807 (85.4)
Private	29 (9.2)	33 (10.5)	50 (15.9)	112 (11.9)
Self-pay	6 (1.9)	7 (2.2)	13 (4.1)	26 (2.8)
Childhood vaccine refusal				
No past MMR and DTaP	2 (0.6)	4 (1.3)	3 (1.0)	9 (1.0)
Patient lifetime historical no-show rate, mean (SD)	0.14 (0.28)	0.18 (0.33)	0.17 (0.33)	0.16 (0.32)
Time since last WCC, mean (range)	17.0 (12.0-24.0)	17.2 (12.0-24.0)	16.9 (12.0-24.0)	17.0 (12.0-24.0)

Abbreviations: DTaP, diphtheria and tetanus toxoids and acellular pertussis; MMR, measles, mumps, and rubella; WCC, well child care.

### Intention-to-Treat Analyses

**Figure 2.**
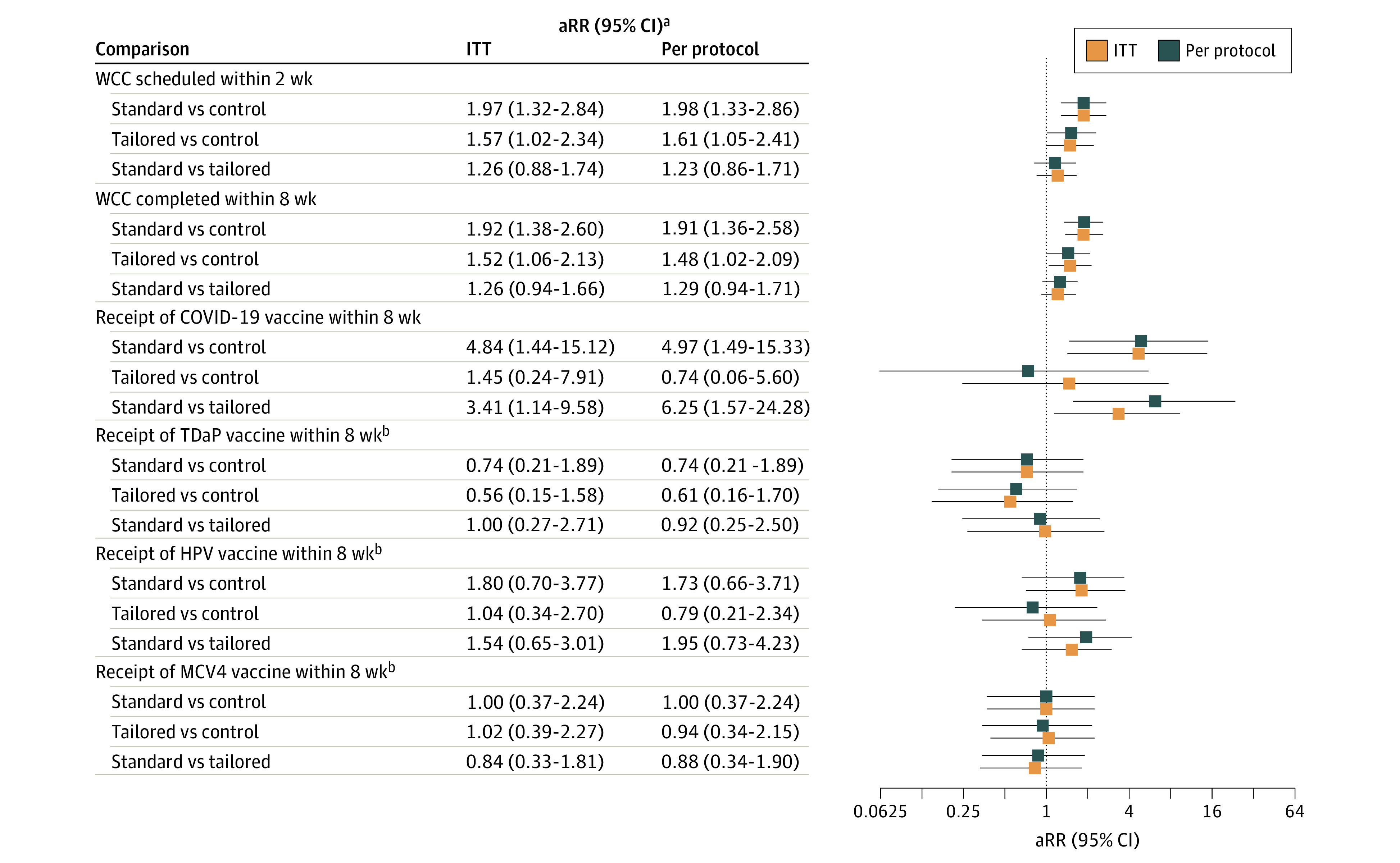
Adjusted Risk Ratios (aRRs) for Intention-to-Treat (ITT) and Per Protocol Analyses The intention-to-treat and per protocol analyses were adjusted for insurance type, as those in the control group appeared to have fewer patients with public insurance compared with the standard and tailored message groups. HPV indicates human papillomavirus; MCV4, meningococcal conjugate vaccine; TDaP, tetanus, diphtheria, acellular pertussis; and WCC, well child care. ^a^The intent-to-treat and per-protocol analyses were adjusted for insurance type, as those in the control group appeared to have less patients with public insurance compared with the standard and tailored message groups. ^b^Analysis includes all patients eligible to receive this vaccine during the 8-week study period.

There were 58 of 315 patients (18.4%; adjusted RR [aRR], 1.97; 95% CI, 1.32-2.84) in the standard message, 47 of 315 patients (14.9%; aRR, 1.57; 95% CI, 1.02-2.34) in the tailored message, and 30 of 315 patients (9.5%) in the control groups who scheduled a WCC visit within 2 weeks (Table 2). The standard message and tailored message groups had higher rates of scheduling a WCC compared with the control group (Figure 2). Well child care visit completion rates within 8 weeks were also higher in the standard message (24.1%; aRR, 1.92; 95% CI, 1.38-2.60) and tailored message (19.4%; aRR, 1.52; 95% CI, 1.06-2.13) groups compared with the control group (12.7%). Among those eligible to receive the COVID-19 vaccine (only approved for patients aged ≥12 years old at the time of the study), 14 patients (16.7%) in the standard message group, 4 patients (4.8%; aRR, 3.41; 95% CI, 1.14-9.58) in the tailored message group, and 3 patients (3.7%; aRR, 4.84; 95% CI, 1.44-15.12) in the control group received the vaccine within 8 weeks. The standard message group had higher rates of receiving the COVID-19 vaccine compared with the tailored message and control groups. Among those eligible to receive the DTaP, HPV, and/or MCV4 vaccines during the study period, the rates of receiving these vaccines within 8 weeks did not differ significantly between groups (Table 2). Rates of receiving these vaccines within 8 weeks across the standard message group vs tailored message group vs control group were as follows: DTaP, 19% vs 18% vs 25%; HPV, 27% vs 17% vs 14%; and MCV4, 19% vs 21% vs 18%. Due to high baseline rates, by the end of the study, most participants in the sample had received the DTaP (76%) and the first dose of MCV4 (75%) vaccines and the first dose of the HPV (62%) vaccine. There was no significant difference in the mean (SD) time since last WCC between those who did (17.16 [3.93] months) and did not (17.18 [4.08] months) complete a WCC in the tailored message group (*P* = .98).

**Table 2.** Intention-to-Treat Rates of Completion

Outcome	No./total No. (%)			
	Standard message	Tailored message	Control group	Total
WCC scheduled within 2 wk	58/315 (18.4)	47/315 (14.9)	30/315 (9.5)	135/945 (14.3)
WCC completed within 8 wk	76/315 (24.1)	61/315 (19.4)	40/315 (12.7)	177/945 (18.7)
Receipt of COVID vaccination within 8 wk^a^	14/84 (16.7)	4/83 (4.8)	3/82 (3.7)	21/249 (8.4)
Receipt of DTaP within 8 wk^a^	6/32 (30.0)	6/34 (30.0)	8/32 (40.0)	20/98 (20.4)
Receipt of HPV within 8 wk^a^	13/49 (44.8)	9/52 (31.0)	7/50 (24.1)	29/151 (19.2)
Receipt of MCV4 within 8 wk^a^	10/54 (32.3)	12/57 (38.7)	9/49 (29.0)	31/160 (19.4)

Abbreviations: HPV, human papillomavirus vaccine; MCV4, meningococcal conjugate vaccine; DTaP, diphtheria and tetanus toxoids and acellular pertussis; WCC, well child care.

^a^ Analysis includes all patients eligible to receive this vaccine during the 8-week study period.

### Sensitivity Analysis

We identified 54 of 315 patients in the control group (17%) who shared the same telephone number and/or account proxy with 1 or more patients included in a message group, indicating participants were likely siblings. Excluding patients in the control group with siblings in a message group did not change the intention-to-treat pattern of the results.

### Process Measures

Reasons for not sending the intended intervention on both days included scheduled a WCC visit after randomization but before sending first message (standard: n = 1 of 315; tailored: n = 29 of 315), scheduled a WCC visit after the first message (standard: n = 25 of 315; tailored: n = 19 of 215), and patient portal account inactivated after randomization but before message sent (standard: n = 2 of 315). Delivery of at least 1 patient portal message had high success rates across the standard message (99%) and tailored message (91%) groups (Figure 1). Therefore, our per-protocol analysis included a total of 913 patients who received the intended intervention.

### Per Protocol Analyses

Overall, the pattern of results was the same as the intention-to-treat analysis (Table 3 and Figure 2), with all group differences previously observed in intention-to-treat analysis remaining significant and in the same direction. Sensitivity analysis excluding patients in the control group with siblings in a message group did not change the pattern of results.

**Table 3.** Per Protocol Rates of Completion, No. (%)

Outcome	No./total No. (%)			
	Standard message	Tailored message	Control group	Total
WCC scheduled within 2 wk	59/312 (18.9)	44/286 (15.4)	30/315 (9.5)	133/913 (14.6)
WCC completed within 8 wk	76/312 (24.4)	54/286 (18.9)	40/315 (12.7)	170/913 (18.6)
Receipt of COVID vaccination within 8 wk^a^	14/81 (17.3)	2/74 (2.7)	3/82 (3.7)	19/237 (8.0)
Receipt of DTaP within 8 wk^a^	6/32 (30.0)	6/31 (30.0)	8/32 (40.0)	20/95 (21.1)
Receipt of HPV within 8 wk^a^	12/47 (48.0)	6/46 (24.0)	7/50 (28.0)	25/143 (17.5)
Receipt of MCV4 within 8 wk^a^	10/54 (33.3)	11/53 (36.7)	9/49 (30.0)	30/156 (19.2)

Abbreviations: HPV, human papillomavirus vaccine; MCV4, meningococcal conjugate vaccine; DTaP, diphtheria and tetanus toxoids and acellular pertussis; WCC, well child care.

^a^ Analysis includes all patients eligible to receive this vaccine during the 8-week study period.

## Discussion

In this randomized clinical trial of patient portal outreach messages among predominantly non-Hispanic Black children overdue for well care with active portal accounts, the standard message and tailored message both positively affected WCC scheduling and completion. The standard message group outperformed the tailored message and control groups on COVID-19 vaccine completion among eligible patients.

There was a significant increase in the scheduling and completion of appointments after receiving the standard and tailored message compared with no message, indicating that simple outreach nudges via patient portal may prompt action. The standard message group had higher rates of receiving COVID-19 vaccination within 8 weeks, suggesting that messages that reengage patients subsequently provide opportunities to promote healthy behaviors, such as vaccine acceptance. A focus group found that families with patient portal accounts prefer straightforward, brief, and user-friendly messages.^19^ This indication of preference may help explain our finding that the standard message outperformed the tailored message on the COVID-19 vaccine completion outcome. Families may have been distracted by the additional information related to the child’s age and date of last WCC in the tailored message. Including the date of the last WCC should have made salient that the child was overdue for a WCC, addressing distortions of time perception experienced by many during the pandemic.^27,28,29^ Yet some families may have perceived that differently if unfamiliar with the recommended periodicity schedule. In addition, we did not find a significant difference in time since the last WCC on WCC completion within 8 weeks among patients in the tailored message group. Further research is needed to assess how varying message content impacts outcome completion.

To our knowledge, no previous studies have determined the effect of patient portal messaging to promote WCC scheduling and completion among pediatric patients overdue for care. Nudge health maintenance reminders sent via the patient portal were associated with increased appointment scheduling rates among adults aged 65 years and older; however, no significant differences were observed between the control and nudge groups on wellness visit completion rates.^24^ Success rates for studies using text messaging or telephone call interventions to promote completion of well child appointments range from 14% to 72%.^13,14,32,33,34^ Differences in rates of WCC completion compared with these studies may be due to (1) longer duration of follow-up in earlier studies, ranging from 6 to 18 months; (2) more than half the sample in previous studies had a WCC in the 12 months before intervention vs none in ours; or (3) earlier studies used higher intensity interventions.^13,32,34^ Using patient portals offers an additional layer in the approach to engage families, as outreach attempts need to be varied, especially in marginalized populations.^9^ Future qualitative research should be done to (1) incorporate multiple communication modes, such as videos and storytelling; (2) offer and encourage choice; (3) develop patient-centered messages by cocreating with families; and (4) use interventions that leverage effective community partnerships and trust.

Past research on patient portal messages has largely examined its use among adult, non-Hispanic White, and privately insured populations.^18,19,20,21,22,23^ In contrast, our study examined the use of patient portal messages among predominantly low-income non-Hispanic Black pediatric patients, a traditionally marginalized and harder to reach population. We conducted a separate trial with patients with an inactive EHR portal account to examine text and telephone reminder messages in that population, and rates of WCC completion were lower, ranging from 10% to 14%.^35^ Demonstrating an increased rate of completing WCC visits suggests that patient portal messaging may be a valid way of reaching populations that have been historically marginalized. Effects may be even higher in populations that are traditionally more likely to use EHR portals or in nonpandemic times.

Compared with those included in our study, patients excluded due to lack of an active portal account differed on race (higher proportion of Black children), age (younger), and insurance (higher proportion with self-pay and publicly insured children). Previous research highlights substantial disparities in pediatric patient portal use among Medicaid-enrolled, low-income, and Black and Latinx patients.^16,17,25,36^ Others attribute these differences to less internet access or computers,^37^ yet cell phone use and desire to receive health information via cell phones has been found to be prevalent among low-income populations.^38^ Past research has found that White patients are more likely to receive access codes to activate their portal account compared with Black or Latinx patients.^39,40^ In our practices, portal enrollment opportunities are offered at appointments, so patients eligible for the present study (ie, no WCC visit in the past 12 months) had fewer recent opportunities to enroll than children seen frequently. It is unclear whether the disparities related to patient portal use relates to discrimination by the medical system, health literacy barriers, or differences in how families use the internet. Regardless, more research is needed to understand and promote patient portal use among vulnerable populations.^41^

### Strengths and Limitations

Study strengths include the use of a clinically relevant, presumably low-cost and widely available intervention; a large, historically marginalized, at-risk sample; ascertainment of important outcomes; and intention-to-treat design. Our study also has limitations. It was conducted in academic primary care practices serving low-income, predominantly Black patients, which limits the generalizability of results, but complements past reports^26^ by focusing on a marginalized group. Due to challenges related to attribution and time since the last appointment, it is possible some children were no longer current patients. The groups in this study were contaminated with children in the same household (ie, same telephone number and/or caregiver listed) being allocated to different groups. This led to some members of the control group being exposed to messages, although sensitivity analyses show this did not impact our estimates. We were unable to accurately determine the date messages were first read, as this variable is updated every time the message is viewed. Our messaging interventions were developed by a study team that does not reflect the racial composition of our patient population. We acknowledge that outreach messages we view as helpful may still lack relevance to the lived experiences of marginalized populations. Although we received feedback from parents and outreach medical assistants on message content, future effort is needed to seek deeper engagement in intervention development.

## Conclusions

In this randomized clinical trial, outreach messages delivered via EHR patient portals increased the rates of scheduling and completing overdue WCC visits and receiving the COVID-19 vaccine. Additional efforts are needed to reengage at-risk populations and recover preventive services missed during the pandemic.

## Notes

Supplement 1.
Trial Protocol

Supplement 2. **eTable 1.** Unadjusted Risk Ratios (RR) for Intent-to-Treat and Per Protocol Analyses **eTable 2.** Example of Patient Portal Outreach Messages

Supplement 3.
Data Sharing Statement

## References

[1] Bode SM, Gowda C, Mangini M, Kemper AR. COVID-19 and primary measles vaccination rates in a large primary care network. Pediatrics. 2021;147(1):e2020035576. doi:10.1542/peds.2020-035576 33214332

[2] Dinleyici EC, Borrow R, Safadi MAP, van Damme P, Munoz FM. Vaccines and routine immunization strategies during the COVID-19 pandemic. Hum Vaccin Immunother. 2021;17(2):400-407. doi:10.1080/21645515.2020.1804776 32845739 PMC7899627

[3] Patel Murthy B, Zell E, Kirtland K, . Impact of the COVID-19 pandemic on administration of selected routine childhood and adolescent vaccinations—10 US jurisdictions, March-September 2020. MMWR Morb Mortal Wkly Rep. 2021;70(23):840-845. doi:10.15585/mmwr.mm7023a2 34111058 PMC8191867

[4] Santoli JM, Lindley MC, DeSilva MB, . Effects of the COVID-19 pandemic on routine pediatric vaccine ordering and administration—United States, 2020. MMWR Morb Mortal Wkly Rep. 2020;69(19):591-593. doi:10.15585/mmwr.mm6919e2 32407298

[5] DeSilva MB, Haapala J, Vazquez-Benitez G, . Association of the COVID-19 pandemic with routine childhood vaccination rates and proportion up to date with vaccinations across 8 US health systems in the Vaccine Safety Datalink. JAMA Pediatr. 2022;176(1):68-77. doi:10.1001/jamapediatrics.2021.4251 34617975 PMC8498937

[6] Ackerson BK, Sy LS, Glenn SC, . Pediatric vaccination during the COVID-19 pandemic. Pediatrics. 2021;148(1):e2020047092. doi:10.1542/peds.2020-047092 33858983

[7] Okoro O, Kennedy J, Simmons G Jr, . Exploring the scope and dimensions of vaccine hesitancy and resistance to enhance COVID-19 vaccination in Black communities. J Racial Ethn Health Disparities. 2021:1-14. doi:10.1007/s40615-021-01150-0 34553340 PMC8457035

[8] Momplaisir F, Haynes N, Nkwihoreze H, Nelson M, Werner RM, Jemmott J. Understanding drivers of coronavirus disease 2019 vaccine hesitancy among Blacks. Clin Infect Dis. 2021;73(10):1784-1789. doi:10.1093/cid/ciab102 33560346 PMC7929035

[9] Balasuriya L, Santilli A, Morone J, . COVID-19 vaccine acceptance and access among Black and Latinx communities. JAMA Netw Open. 2021;4(10):e2128575. doi:10.1001/jamanetworkopen.2021.28575 34643719 PMC8515205

[10] Quinn SC, Andrasik MP. Addressing vaccine hesitancy in BIPOC communities—toward trustworthiness, partnership, and reciprocity. N Engl J Med. 2021;385(2):97-100. doi:10.1056/NEJMp2103104 33789007

[11] Jacobson Vann JC, Jacobson RM, Coyne-Beasley T, Asafu-Adjei JK, Szilagyi PG. Patient reminder and recall interventions to improve immunization rates. Cochrane Database Syst Rev. 2018;1:CD003941. doi:10.1002/14651858.CD003941.pub3 29342498 PMC6491344

[12] Szilagyi PG, Humiston SG, Gallivan S, Albertin C, Sandler M, Blumkin A. Effectiveness of a citywide patient immunization navigator program on improving adolescent immunizations and preventive care visit rates. Arch Pediatr Adolesc Med. 2011;165(6):547-553. doi:10.1001/archpediatrics.2011.73 21646588

[13] Suh CA, Saville A, Daley MF, . Effectiveness and net cost of reminder/recall for adolescent immunizations. Pediatrics. 2012;129(6):e1437-e1445. doi:10.1542/peds.2011-1714 22566415

[14] Szilagyi PG, Albertin C, Humiston SG, . A randomized trial of the effect of centralized reminder/recall on immunizations and preventive care visits for adolescents. Acad Pediatr. 2013;13(3):204-213. doi:10.1016/j.acap.2013.01.002 23510607 PMC4594853

[15] Masterman M, Cronin RM, Davis SE, Shenson JA, Jackson GP. Adoption of secure messaging in a patient portal across pediatric specialties. AMIA Annu Symp Proc. 2017;2016:1930-1939. 28269952 PMC5333207

[16] Anthony DL, Campos-Castillo C, Lim PS. Who isn’t using patient portals and why? evidence and implications from a national sample of US adults. Health Aff (Millwood). 2018;37(12):1948-1954. doi:10.1377/hlthaff.2018.05117 30633673

[17] Turner K, Clary A, Hong Y-R, Alishahi Tabriz A, Shea CM. Patient portal barriers and group differences: cross-sectional national survey study. J Med internet Res. 2020;22(9):e18870. doi:10.2196/18870 32940620 PMC7530687

[18] Lerner C, Albertin C, Casillas A, . Patient portal reminders for pediatric influenza vaccinations: a randomized clinical trial. Pediatrics. 2021;148(2):e2020048413. doi:10.1542/peds.2020-048413 34321338 PMC8669575

[19] Szilagyi PG, Albertin C, Casillas A, . Effect of patient portal reminders sent by a health care system on influenza vaccination rates: a randomized clinical trial. JAMA Intern Med. 2020;180(7):962-970. doi:10.1001/jamainternmed.2020.1602 32421168 PMC7235900

[20] Ueberroth BE, Labonte HR, Wallace MR. Impact of patient portal messaging reminders with self-scheduling option on influenza vaccination rates: a prospective, randomized trial. J Gen Intern Med. 2022;37(6):1394-1399. doi:10.1007/s11606-021-06941-z 34131878 PMC8205315

[21] Wijesundara JG, Ito Fukunaga M, Ogarek J, . Electronic health record portal messages and interactive voice response calls to improve rates of early season influenza vaccination: randomized controlled trial. J Med internet Res. 2020;22(9):e16373. doi:10.2196/16373 32975529 PMC7547389

[22] Cutrona SL, Golden JG, Goff SL, . Improving rates of outpatient influenza vaccination through EHR portal messages and interactive automated calls: a randomized controlled trial. J Gen Intern Med. 2018;33(5):659-667. doi:10.1007/s11606-017-4266-9 29383550 PMC5910339

[23] Szilagyi PG, Albertin CS, Casillas A, . Effect of personalized messages sent by a health system’s patient portal on influenza vaccination rates: a randomized clinical trial. J Gen Intern Med. 2022;37(3):615-623. doi:10.1007/s11606-021-07023-w 34472020 PMC8858355

[24] Liang S-Y, Stults CD, Jones VG, . Effects of behavioral economics-based messaging on appointment scheduling through patient portals and appointment completion: observational study. JMIR Hum Factors. 2022;9(1):e34090. doi:10.2196/34090 35353051 PMC9008532

[25] Ketterer T, West DW, Sanders VP, Hossain J, Kondo MC, Sharif I. Correlates of patient portal enrollment and activation in primary care pediatrics. Acad Pediatr. 2013;13(3):264-271. doi:10.1016/j.acap.2013.02.002 23680344

[26] Szilagyi PG, Valderrama R, Vangala S, . Pediatric patient portal use in one health system. J Am Med Inform Assoc. 2020;27(3):444-448. doi:10.1093/jamia/ocz203 31841146 PMC7647250

[27] Holman EA, Jones NM, Garfin DR, Silver RC. Distortions in time perception during collective trauma: Insights from a national longitudinal study during the COVID-19 pandemic. Psychol Trauma. 2022. doi:10.1037/tra0001326 35925689 PMC9898469

[28] Ogden RS. The passage of time during the UK Covid-19 lockdown. PLoS One. 2020;15(7):e0235871. doi:10.1371/journal.pone.0235871 32628735 PMC7337311

[29] Grondin S, Mendoza-Duran E, Rioux P-A. Pandemic, quarantine, and psychological time. Front Psychol. 2020;11:581036. doi:10.3389/fpsyg.2020.581036 33192897 PMC7641621

[30] Center for Clinical and Translational Science and Training. COVID-19 data and research projects. Accessed November 5, 2021. https://www.cctst.org/covid19

[31] Zhang J, Yu KF. What’s the relative risk? a method of correcting the odds ratio in cohort studies of common outcomes. JAMA. 1998;280(19):1690-1691. doi:10.1001/jama.280.19.1690 9832001

[32] Marron RL, Lanphear BP, Kouides R, Dudman L, Manchester RA, Christy C. Efficacy of informational letters on hepatitis B immunization rates in university students. J Am Coll Health. 1998;47(3):123-127. doi:10.1080/07448489809595632 9830818

[33] Staras SA, Vadaparampil ST, Livingston MD, Thompson LA, Sanders AH, Shenkman EA. Increasing human papillomavirus vaccine initiation among publicly insured Florida adolescents. J Adolesc Health. 2015;56(5)(suppl):S40-S46. doi:10.1016/j.jadohealth.2014.11.024 25863554 PMC4394203

[34] Szilagyi PG, Schaffer S, Barth R, . Effect of telephone reminder/recall on adolescent immunization and preventive visits: results from a randomized clinical trial. Arch Pediatr Adolesc Med. 2006;160(2):157-163. doi:10.1001/archpedi.160.2.157 16461871

[35] Burkhardt MC, Berset AE, Xu Y, Mescher A, Brinkman WB. Effect of outreach messages on adolescent well child visits and COVID-19 vaccine rates: an RCT. J Pediatr.S0022-3476(22)00857-5. doi:10.1016/j.jpeds.2022.09.035 36202236 PMC9529346

[36] LeLaurin JH, Nguyen OT, Thompson LA, . Disparities in pediatric patient portal activation and feature use. JAMIA Open. 2021;4(3):ooab086. doi:10.1093/jamiaopen/ooab086 34604712 PMC8480543

[37] Sarkar U, Karter AJ, Liu JY, . The literacy divide: health literacy and the use of an internet-based patient portal in an integrated health system-results from the diabetes study of northern California (DISTANCE). J Health Commun. 2010;15(suppl 2):183-196. doi:10.1080/10810730.2010.499988 20845203 PMC3014858

[38] Kwon NS, Colucci A, Gulati R, . A survey of the prevalence of cell phones capable of receiving health information among patients presenting to an urban emergency department. J Emerg Med. 2013;44(4):875-888. doi:10.1016/j.jemermed.2012.09.041 23321292

[39] Ancker JS, Barrón Y, Rockoff ML, . Use of an electronic patient portal among disadvantaged populations. J Gen Intern Med. 2011;26(10):1117-1123. doi:10.1007/s11606-011-1749-y 21647748 PMC3181304

[40] Ancker JS, Nosal S, Hauser D, Way C, Calman N. Access policy and the digital divide in patient access to medical records. Health Policy Technol. 2017;6(1):3-11. doi:10.1016/j.hlpt.2016.11.004

[41] George S, Duran N, Norris K. A systematic review of barriers and facilitators to minority research participation among African Americans, Latinos, Asian Americans, and Pacific Islanders. Am J Public Health. 2014;104(2):e16-e31. doi:10.2105/AJPH.2013.301706 24328648 PMC3935672

