# Supplementary material for: Effect of Electronic Outreach Using Patient Portal Messages on Well Child Care Visit Completion: A Randomized Clinical Trial
Source: JAMA Netw Open. 2022 Nov 18;5(11):e2242853. doi: 10.1001/jamanetworkopen.2022.42853 (PMC9675005; doi:10.1001/jamanetworkopen.2022.42853)
Supplement: Supplement 1. — Trial Protocol [file jamanetwopen-e2242853-s001.pdf]

Official Title of the study: **Effect of Patient Portal Outreach Messages on Well Child Care Visit Catch Up: A Randomized Clinical Trial**

NCT number: **NCT04994691**

Document name: **Study Protocol**

Date of the document: **6/27/2022**

Redesigning Outreach Post-COVID-19 in Academic Pediatric Primary Care

William Brinkman, MD, MS
Professor of Pediatrics
Division Director
General & Community Pediatrics
Cincinnati Children's Hospital Medical Center
3333 Burnet Ave.
Cincinnati, OH 45229
Bill.Brinkman@cchmc.org

Anne Mescher, BSN, MSN
Clinical Manager
General & Community Pediatrics
Cincinnati Children's Hospital Medical Center
3333 Burnet Ave.
Cincinnati, OH 45229
Anne.Mescher@cchmc.org

Mary Carol Burkhardt, MD, MHA
Associate Division Director, Primary Care
General & Community Pediatrics
Cincinnati Children's Hospital Medical Center
3333 Burnet Ave.
Cincinnati, OH 45229
Mary.Burkhardt@cchmc.org

Tasha Johnson, MD
Staff Physician 1
General & Community Pediatrics
Cincinnati Children's Hospital Medical Center
3333 Burnet Ave.
Cincinnati, OH 45229
Tasha.Johnson@cchmc.org

Anne Berset, BA
Clinical Research Coordinator III
General & Community Pediatrics
Cincinnati Children’s Hospital Medical Center
3333 Burnet Ave.
Cincinnati, OH 45229
Anne.berset@cchmc.org

Yingying Xu, PhD
Senior Biostatistician
General & Community Pediatrics
Cincinnati Children’s Hospital Medical Center
3333 Burnet Ave.
Cincinnati, OH 45229
Yingying.Xu@cchmc.org

1. **Objective**

Our objective is to determine the effectiveness of varied outreach methods (e.g. automated reminder calls/text messages with or without personalized calls/texts, MyChart messaging) to children age 12-14 months, 4 years old, or 6-17 years old who are due for a WCC visit and don't have one scheduled in the next 45 days on the outcomes of appointment scheduling, appointment completion, and receipt of the MMR vaccination.

Background

The Cincinnati Children's Hospital Medical Center Primary Care offices serve as the medical home for over 33,000 children. The Pediatric Primary Care Center (PPCC), Hopple Street Health Center (HPC) and Fairfield Primary Care (FPC) serves an underserved population. Annually, these practices provide over 60,000 visits. Traditional outreach efforts in these offices have included reminder phone calls and/or texts when patients were overdue for well child care (WCC). These efforts were time and resource intensive, as they required personnel to manually complete and document attempts. Additionally, due to high resource needs to accomplish such efforts, there has been no outreach to those 6 years and older.

Numerous studies exist speaking to the efficacy of automated reminder (i.e. notifying parents of needed immunizations) and recall (i.e. notifying parents of overdue immunizations) systems, including text messaging systems.[1,2,3] Other studies show that the combination of texting and calls proves even more successful in reaching patients.[3] These systems are more cost effective and efficient. Differences in the timing of reminders to schedule, as well as the message itself, has been shown to make a difference in the outcomes of reaching patients or scheduling appointments.[4] However, there remains a need for research that identifies the degree to which combinations of patient reminder systems improve outcomes, especially the outcome of on-time immunization, and how these approaches differ by age of child.[1]

Following the COVID-19 pandemic shut-down in which thousands of well child appointments were cancelled or delayed, the importance of a systematic outreach process became more critical. The traditional process is non-standardized and the efficacy of the work is both unknown and expensive. Our objective is to determine the effectiveness of varied outreach methods (e.g. automated reminder calls/text messages with or without personalized calls/texts, MyChart messaging) to children age 12-14 months, 4 years old, or 6-17 years old who are due for a WCC visit and don't have one scheduled in the next 45 days on the outcomes of appointment scheduling, appointment completion, and receipt of the MMR vaccination.

2. **Design/Method**

To achieve our objective, we will conduct a 2 × 2 factorial randomized controlled trial. Randomization is justified because 1) it is a rigorous approach to answer our question and 2) we do not have the resources or appointment availability to personally contact every patient who is eligible and have them schedule immediately.

Patient Population:

Patients are eligible for inclusion in this study if they are in one of four eligible groups:

**12-14 months AND**	**4 years AND**
• doesn't have a well visit scheduled in the next 45 days<br>• have not yet received 12-month immunizations<br>• have English and/or Spanish as preferred language	• doesn't have a well visit scheduled in the next 45 days<br>• has not had a well child visit in 365 days<br>• have English and/or Spanish as preferred language

**6-11 years AND**	**12-17 years AND**
• doesn't have a well visit scheduled in the next 45 days	• doesn't have a well visit scheduled in the next 45 days

• has not had a well child visit > 365 days<br>• have English and/or Spanish as preferred language	• has not had a well child visit in 365 days<br>• have English and/or Spanish as preferred language

Factors under study (levels):

Personal Contacts (12-14 month & 4-year-olds) Trial:
Number of automated messages sent prior to any personal contact attempts (1 vs. 2)
Number of personal contact attempts made by medical assistant (0 vs. 1)

	**1 automated message sent**	**2 automated messages sent**
**0 personal contact attempt**		
**1 personal contact attempt**		

MyChart Trial:
Families enrolled in MyChart will be selected and randomized into the following groups:

	**no message**	**1 MyChart message sent**	**2 MyChart messages (e.g. Mon and Thurs)**
**Low Tailored Message**			
**High Tailored Message**			

Number of messages (0 vs. 1 vs. 2); Type of messages (low tailored message with name only vs. high tailored message with date of last WCC and age included)

Televox (age 6-11yo) Trial:
Patients will be randomized into the following three groups. Patients with MyChart are excluded:

**no message**	**Low tailored Message**<br>2 automated standard messages sent (e.g. Thu, Fri)	**High Tailored Message**<br>2 automated tailored messages sent (e.g. Thu, Fri)

Televox (age 12-17yo) Trial:
Patients will be randomized into the following three groups:

**no message**	**Low Tailored Message**<br>2 automated tailored messages sent (e.g. Thu, Fri)	**COVID Tailored Message**<br>2 automated messages sent (e.g. Thu, Fri)

<u>Automated Message</u>: Parents indicate their preferred mode of reminder (e.g. call or text message) when they schedule appointments. Cincinnati Children's uses a HIPAA compliant platform called TeleVox to send automated calls and text reminders to parents according to their preferences which are recorded in the Electronic Health Record (EHR).

Here is the script for **low tailored automated messages** that will be sent via call or text:

Clinic	Message 1*	Message 2*
HPC	Hi! Hopple misses you! (INSERT FIRST NAME) is due for a checkup. Please call 513-517-2000, option 1 to schedule a visit as soon as possible.	Hi! If you haven't already, please call 513-517-2000, option 1 to schedule (INSERT FIRST NAME)'s upcoming visit at Hopple.
FPC	Hi! Fairfield misses you! (INSERT FIRST NAME) is due for a checkup. Please call 513-636-8259, option 1 to schedule a visit as soon as possible.	Hi! If you haven't already, please call 513-636-8259, option 1 to schedule (INSERT FIRST NAME)'s upcoming visit at Fairfield.
PPC	Hi! PPC misses you! (INSERT FIRST NAME) is due for a checkup. Please call 513-636-7722, option 1 to schedule a visit or walk in today.	Hi! If you haven't already, please call 513-636-7722, option 1 to schedule (INSERT FIRST NAME)'s upcoming visit at PPC or walk in today.

*Message Scripts have been translated into Spanish by Interpreter Services

This is the script for **high tailored automated messages** that will be sent via call or text:

Clinic	Message 1*	Message 2*
HPC	(INSERT FIRST NAME) was seen for a checkup at Hopple on XX/XX/XXXX & is due for a (INSERT CURRENT AGE) checkup. Please call 513-517-2000 opt 1.	If you haven't, please call 513-517-2000 opt 1 to schedule (INSERT FIRST NAME)'s (INSERT CURRENT AGE) checkup at Hopple. Stay up to date!
FPC	(INSERT FIRST NAME) was seen for a checkup at Fairfield on XX/XX/XXXX & is due for a (INSERT CURRENT AGE) checkup. Please call 513-636-8259 opt 1.	If you haven't, please call 513-636-8259 opt 1 to schedule (INSERT FIRST NAME)'s (INSERT CURRENT AGE) checkup at Fairfield. Stay up to date!
PPC	(INSERT FIRST NAME) was seen for a checkup at PPC on XX/XX/XXXX & is due for a (INSERT CURRENT AGE) checkup. Please call 513-636-7722 opt 1 to schedule or walk in today.	If you haven't, please call 513-636-7722 opt 1 to schedule (INSERT FIRST NAME)'s (INSERT CURRENT AGE) checkup at PPC or walk in today. Stay up to date!

This is the script for **COVID tailored automated messages** that will be sent via call or text:

Clinic	Message 1*	Message 2*
HPC	Hi! Hopple misses you! (INSERT FIRST NAME) is due for a checkup. Please call 513-517-2000, option 1 to schedule a visit as soon as possible. COVID vaccine is available if interested.	Hi! If you haven't already, please call 513-517-2000, option 1 to schedule (INSERT FIRST NAME)'s upcoming visit at Hopple. COVID vaccine is available if interested.
FPC	Hi! Fairfield misses you! (INSERT FIRST NAME) is due for a checkup. Please call 513-636-8259, option 1 to schedule a visit as soon as possible. COVID vaccine is available if interested.	Hi! If you haven't already, please call 513-636-8259, option 1 to schedule (INSERT FIRST NAME)'s upcoming visit at Fairfield. COVID vaccine is available if interested.
PPC	Hi! PPC misses you! (INSERT FIRST NAME) is due for a checkup. Please call 513-636-7722, option 1 to schedule a visit or walk in today. COVID vaccine is available if interested.	Hi! If you haven't already, please call 513-636-7722, option 1 to schedule (INSERT FIRST NAME)'s upcoming visit at PPC or walk in today. COVID vaccine is available if interested.

*Message Scripts have been translated into Spanish by Interpreter Services

<u>Personal Contact Attempts</u>: Medical assistant(s) will use the following message/script to personally contact families via text message and then phone call.

Clinic	Text Message*	Call Script*

HPC	Hi, this is [Staff First Name] from Hopple St. Health Center. [Pt first name] is due for a check up. I'll call in a day or two to help schedule that appointment or you can call 513-517-2000, option 1 to schedule.	Hi, this is [Staff First Name] from Hopple St. Health Center. I noticed that [Child's First Name] is due for a check up. Can I schedule that for you right now?<br><br>Alternate script for leaving a voice message: Hi, this is [Staff First Name] from Hopple St. Health Center. I noticed that [Child's First Name] is due for a check up. Sorry I missed you. Please call 513-517-2000, option 1 to schedule your appointment.
FPC	Hi, this is [Staff First Name] from Fairfield. [Pt first name] is due for a check up. I'll call in a day or two to help schedule that appointment or you can call 513-517-2000, option 1 to schedule.	Hi, this is [First Name] from Fairfield. I noticed that [Child's First Name] is due for a check up. Can I schedule that for your right now?<br><br>Alternate script for leaving a voice message: Hi, this is [First Name] from Fairfield. I noticed that [Child's First Name] is due for a check up. Sorry I missed you. Please call 513-636-8259, option 1 to schedule your appointment.
PPC	Hi, this is [Staff First Name] from PPC. [Pt first name] is due for a check up. I'll call in a day or two to help schedule that appointment or you can walk in today to have your child seen! You can also call 513-636-7722, option 1 to schedule.	Hi, this is [First Name] from PPC. I noticed that [Child's First Name] is due for a check up. Can I schedule that for your right now?<br><br>Alternate script for leaving a voice message: Hi, this is [First Name] from PPC. I noticed that [Child's First Name] is due for a check up. Sorry I missed you. Please call 513-636-7722, option 1 to schedule your appointment or walk in today to have your child seen.

*Scripts have been translated into Spanish versions by Interpreter Services

Response variables (outcomes):
- Appointment **was scheduled** within 2 weeks of intervention (Y/N) (date of actual appointment may be greater than 2 weeks from intervention)
- Appointment **was completed** within 8 weeks of intervention (Y/N). Appointment completed within 8 weeks of randomization will be used for group who does not receive any intervention.
- (Primary Outcome) Receipt of MMR by 15 months of age or receipt of 2nd MMR dose by 3 months after randomization (Y/N)
- Receipt of 1st COVID vaccination (ages 12-17 years) within 8 weeks of first intervention (Y/N)
- Family member receipt of COVID vaccination in our health system within 8 weeks of first intervention

Blocking: (controlling for factors that might influence the outcome)
- Age (12-14 months, 4 years)
- Clinic location (PPC, HPC, FPC)

Personal Contacts (12-14 months & 4-year-olds) Trial:
Randomization: Eligible patient will be identified by running a report in the electronic health record (EHR). We will run this report, stratified by clinic location, and randomly assign patients into one of the four groups created by our 2 X 2 design. Groups will receive their assigned intervention in one of four successive weeks with the sequence randomly assigned (see table below).

Replication: We will conduct a total of 3 trials, repeating the randomization procedure every 4 weeks to identify children who become eligible: Weeks 1-4 = trial #1, weeks 5-8 = trial #2, weeks 9-12 = trial #3.

	**1 automated message sent**	**2 automated messages sent**
**0 personal contact attempt**	Message sent on Thu (weeks 3, 5, 10)	Message sent on Thu & Fri (weeks 4, 6, 12)
**1 personal contact attempt**	Above plus attempt Mon – Fri of following week (weeks 2, 7, 11)	Above plus attempt Mon – Fri of following week (weeks 1, 8, 9)

MyChart and Televox Trials (6-17 years old):
Randomization: Eligible patients will be identified by running a report in the electronic health record (EHR). We will run this report, stratified by clinic location, and randomly assign patients into one of the study groups as described above. Groups will receive their assigned intervention in one of four successive weeks with the sequence randomly assigned (see table below).

We will randomize children who are eligible and implement interventions for each trial : Weeks 1-3 = Televox Trial 12-17yo, Weeks 4-7 = Televox Trial 6-11yo, Weeks 8-10 = MyChart Trial.

MyChart Trial:

	**no message**	**1 MyChart message sent**	**2 MyChart messages (e.g. Mon and Thurs)**
**Standard Message**	No intervention	Week A	Week B
**Tailored Message**		Week A	Week B

Televox (6-11yo) Trial:

**no message**	**Low Tailored Message**	**High Tailored Message**
	2 automated standard messages sent (e.g. Thu, Fri) (weeks C, C)	2 automated tailored messages sent (e.g. Thu, Fri) (weeks D, D)

Televox (12-17yo) Trial:

**no message**	**Low Tailored Message**	**COVID Tailored Message**
	2 automated tailored messages sent (e.g. Thu, Fri) (week A)	2 automated messages sent (e.g. Thu, Fri) (week B)

Of note, automated messages and/or personal contact attempts will not be completed if the parent schedules an appointment after being randomized but before receiving the intervention.

Covariates for logistic regression: (factors that might influence the outcome if not be balanced across groups)

1. Child age at the time of randomization (rationale: visits are less frequent for children age 4 years)
2. Parent preference for call vs. text mode of contact (rationale: calls may be more likely to get blocked than texts)
3. Parent with activated MyChart account (Y/N) (rationale: not dependent on calling scheduling center to make appointment)

4. Access to appointment: Number of days to 3rd next available appointment at patient's clinic at time their first automated message is sent
5. Patient receipt of any past immunizations (Y/N) (rationale: vaccine refusing parents will be unlikely to change even if they return for well child check)
6. School-age vs. adolescent patient age (rationale: adolescents less likely to get yearly check-ups)
7. Historical no-show rate
8. Community rates of COVID based on 7 day moving average.

Power/Sample Size

Personal Contacts (12-14 month & 4-year-olds) Trial:
We based our hypotheses (see table, below) on a systematic review's pooled estimates of effect size for automated messages and personalized calls finding 37% and 40% of children, respectively, completed immunizations.[1] Because of the significant cost involved in making personal contacts, the minimal difference in outcome that would make it worth the effort is high.

**Hypotheses**	**% of children who receive MMR by 15 months of age**	
	**1 automated message sent**	**2 automated messages sent**
**0 personal attempts**	20% [C]	35% [B]
**1 personal attempt**	50% [A]	65% [AB]

We calculated sample size needed per group to detect a difference for each of the contrasts below with alpha of 0.05 and power of 80%.

**Contrast**	**Sample Size per Group**
A vs C	36
B vs C	136
A vs. B	167
A vs. AB	167
B vs. AB	40
C vs. AB	16

To fully power the 2 X 2 factorial trail, we estimate needing 167 per group X 4 groups = 668 total subjects. There are currently 712 children eligible for the study. More will become eligible every month so we expect to far exceed the required sample size after 3 replications. If needed, we could increase the number of replications of the trial to reach the targeted sample size. We acknowledge that we are not powered to precisely estimate any interactions between interventions.

MyChart Trial:
In hypothesis testing for 0 message vs. 1 message vs. 2 messages, we will do the testing in a fixed sequence - test 2 messages vs. 0 message first, only if it is significant then test 2 messages vs. 1 message, thus the type I error rate of 0.05 is retained. Since the hypothesis testing of 2 messages vs. 0 message would be considered easier to achieve than the hypothesis testing of 2 messages vs. 1 message, the power analysis for this trial should be based on the design of (standard message vs. tailored message) by (1 message vs. 2 messages). We based our hypotheses (see table, below) on previous work indicating approximately 20% WCC appointment completion on automated text reminders. We anticipate that a tailored message increases the WCC appointment completion rate by another 10%.

**Hypotheses**		**% of children complete WCC within 8 weeks**

	No message	1 automated message sent	2 automated messages sent
**Standard Message**	2%	10% C	20% B
**Tailored Message**		20% A	30% AB

We calculated sample size needed per group to detect a difference for each of the contrasts below with alpha of 0.05 and power of 80%.

Contrast	Sample Size per Group
A vs C	199
B vs C	199
A vs. AB	293
B vs. AB	293
C vs. AB	62

We assume no difference between A vs. B and thus not consider powering the comparison between these two groups, but we will explore non-inferiority analysis for this comparison in data analysis. To fully power the above contrasts, we estimate needing 293 per group X 4 groups = 1172 subjects. Adding 293 in the zero message group, we would need a total of 1465 subjects. There are currently 1000 children eligible for the study. More will become eligible every month so we expect to exceed the required sample size after 2 replications. If needed, we could increase the number of replications of this trial to reach the targeted sample size. We acknowledge that we are not powered to precisely estimate any interactions between interventions.

Televox (6-11yo) Trial:
We calculated sample size based on the following hypotheses:

% of children complete WCC within 8 weeks		
**no message**	**2 Low Tailored Messages**	**2 High Tailored Messages**
2%	20%	30%

With alpha of 0.05 and power of 80%, to fully power the contrasts between the above groups, we need 293 per group X 3 groups=879 subjects. Currently there are 2500 kids eligible, so we expect to reach the targeted sample size.

Televox (12-17yo) Trial:
We calculated sample size based on the following hypotheses:

% of children complete WCC within 8 weeks		
**no message**	**2 Low Tailored Messages**	**2 COVID Tailored Messages**
2%	30%	40%

With alpha of 0.05 and power of 80%, to fully power the contrasts between the above groups, we need 356 per group X 3 groups=1068 subjects. Currently there are 1100 kids eligible, so we expect to reach the targeted sample size.

Analysis
We will conduct descriptive analyses to characterize the participants in terms of demographics and other potential covariates in consideration (child age group, parent's preference for call vs. text mode of contact, whether parent

had activated MyChart account, access to appointment, whether patient receipt of any past immunizations, historic no show rate, and community rates of COVID). We will use chi square tests to examine whether participants in the 4 groups are similar in these characteristics.

For the Personal Contact (12-14 mo & 4 years old) trials,we will conduct intent-to-treat analyses using logistic regression models (without adjusting for covariates) for each of our three binary outcomes: completed appointment within 2 weeks, completed appointment within 8 weeks, receipt of MMR by 15 months of age or receipt of 2nd MMR by 3 months after randomization. We will do pair-wise comparison among the 4 groups and adjust p value of each comparison for multiplicity. If participants in the 4 groups differ on potential covariates, we will conduct additional analyses adjusted for these variables.

For the MyChart and Televox Trials (6-17 years old), we will conduct intent-to-treat analyses using logistic regression models (without adjusting for covariates) for each of the binary outcomes: scheduled appointment within 2 weeks, completed appointment within 8 weeks, receipt of 1st dose of COVID vaccination (age 12-17 years) within 8 weeks, family member receipt of COVID vaccination within 8 weeks. We will do pair-wise comparison among the treatment groups and adjust p value of each comparison for multiplicity. If participants differ in potential covariates across treatment groups, we will conduct additional analyses adjusted for these variables. We will explore non-inferiority analysis for comparison the two groups that are expected to have similar appointment scheduled and completion rate in the MyChart trial.

We will also conduct per-protocol analyses according to the interventions actually received by the participants, recognizing that actual intervention received could be different from initial randomized intervention. . For example, a parent allocated to group A might schedule an appointment after receiving the automated message and therefore would not receive a personalized contact attempt. In addition, we may explore potential interaction between the two interventions as a secondary analysis. All analysis will be conducted using SAS version 9.4.

Duration
The study will begin upon IRB approval and continue through December 31, 2022. During this time, we will discuss the significance of data, potential future projects and dissemination of results.

Process of Obtaining Consent
For this study, we request a waiver of consent due to the minimal risk to participants involved. Given the population we are attempting to contact using the various outreach methods has missed appointments, it would not be possible to obtain prospective consent. That said, we will honor parent preferences for how they receive automated messages. Collected data about appointment attendance and MMR vaccination status is already being documented in the EHR, and the collection of data will not affect the rights and welfare of subjects.

We request waiver of consent such that there is (1) potential, minimal, risks to the participant (see below), (2) de-identification of PHI, and (3) participation in the study will not affect current/future care received through CCHMC.

All identifying information will be removed once data collection is complete. Data will be kept securely on a CCHMC computer that is password protected. Any hard copies of data will be locked in a private office.

Facilities and Performance Sites
Data analysis will be done on the Main Campus of CCHMC and at the CCHMC primary care offices (PPCC, HPC, FPC, and SBHCs).

Potential Benefits
The results of this study will aid CCHMC pediatric primary care centers in improving and redesigning an outreach system to children behind on necessary preventative services who have gaps in needed primary care. It will identify the most effective ways to reach, engage, and schedule patients for care when appropriate. This will enable the primary care offices to allocate the appropriate amount of resource to outreach efforts moving forward, ensuring high risk children receive reminders for care.

Potential risks, discomforts, inconveniences, and precautions

The only significant risks are breach of confidentiality and potential discomfort/inconvenience associated with receiving texts or phone calls indicating that care gaps exist for a child. As noted above, personal health information (PHI) will be obtained via the electronic medical record. After obtaining this information, patients will be de-identified in the created database and securely stored.

Risk/Benefit analysis
While immediate benefit to any given individual or family is unknown, this study involves minimal risk.

Cost of Participation

There is no cost for participation.

Payment for Participation
There is no reimbursement for participation.

Request for HIPAA Waiver
The use or disclosure of the PHI involves no more than minimal risk to the privacy of individuals based on the presence of the following elements:
-We plan to protect health information identifiers from improper use and disclosure by secure storage.
-We have an adequate plan to destroy identifiers at the earliest opportunity consistent with conduct of the research.
-We ensure the PHI will not be reused or disclosed to (shared with) any other person or entity, except as required by law, for authorized oversight of the research study, or for other research for which the use or disclosure of the PHI would be permitted under the Privacy Rule.
•This research could not practicably be conducted without the waiver or alteration.
•This research could not practicably be conducted without access to and use of the PHI.

References

1. Jacobson Vann JC, Jacobson RM, Coyne-Beasley T, Asafu-Adjei JK, Szilagyi PG. Patient reminder and recall interventions to improve immunization rates. Cochrane Database of Systematic Reviews 2018, Issue 1. Art. No.: CD003941. DOI: 10.1002/14651858.CD003941.pub3.
2. Arora S, Burner E, Terp S, et al. Improving Attendance at Post–Emergency Department Follow-up Via Automated Text Message Appointment Reminders: A Randomized Controlled Trial. Academic Emergency Medicine 2015;22:31-7.
3. Lin C-L, Mistry N, Boneh J, Li H, Lazebnik R. Text Message Reminders Increase Appointment Adherence in a Pediatric Clinic: A Randomized Controlled Trial. International Journal of Pediatrics 2016;2016:8487378.
4. Reed JL, Huppert JS, Taylor RG, et al. Improving sexually transmitted infection results notification via mobile phone technology. J Adolesc Health 2014;55:690-7.
5. Fortuna RJ, Idris A, Winters P, et al. Get Screened: A Randomized Trial of the Incremental Benefits of Reminders, Recall, and Outreach on Cancer Screening. Journal of General Internal Medicine 2014;29:90-7.

Official Title of the study: **Effect of Patient Portal Outreach Messages on Well Child Care Visit Catch Up: A Randomized Clinical Trial**

NCT number: **NCT04994691**

Document name: **Statistical Analysis Plan**

Date of the document: **6/27/2022**

The results of the Outreach Methods study will be assessed as described in this document. This statistical analysis plan begins with an overview of the key components of the study, including its design, objectives, and outcomes measures. This is followed by a detailed description of the statistical analyses that will be utilized to address study hypotheses.

1. Overview of study: This study used a multi-arm randomized control trial to test the impact of patient portal outreach messages, with and without tailoring, on the scheduling and completion of well child care visits (WCC) and completion of vaccinations. We randomized eligible patients to the Standard Message group, the Tailored Message group, or the Control Group. We generated random allocation sequence stratified by clinic location using block randomization (with a block size of 3). Based on sample size calculations, we planned to enroll a minimum of 879 subjects overall aged 6-17 years old that met eligibility criteria based on data obtained from the electronic health record (EHR) from three academic pediatric primary care practices.

1.1 Study Objective: Test the effectiveness of patient portal outreach messages, with and without tailoring, on the scheduling and completion of well child care visits (WCC) in children due for preventative services.

Working hypothesis: Twenty percent of patients in the Standard Message group, 30% of patients in the Tailored Message group, and 2% of those in the control group will complete a well child care visit within 8 weeks.

1.2 Outcome measures

Primary

- Well child care visit completed within 8 weeks of receiving first intervention
  - Appointment completed (yes or no) based on electronic health record documentation

Secondary

- Well child care visit scheduled within 2 weeks of receiving first intervention
  - Appointment scheduled (yes or no) based on electronic health record documentation
- Child receipt of COVID-19 vaccine within 8 weeks of receiving first intervention among eligible patients (age ≥12 years at the time of the study)
  - COVID-19 vaccine administered (yes or no) based on electronic health record documentation

1.3 Time points of interest

- Scheduled well child care visit (within 2 weeks of receiving first intervention)
- Completed well child care visit (within 8 weeks of receiving first intervention)
- Receipt of COVID-19 vaccine (within 8 weeks of receiving first intervention)

1.4 Data Management

CCHMC CRC will guide data management for this project as described in the study protocol.

1.5 Interim Safety Analyses

No interim analyses are planned for this study.

2. Statistical Reports

2.1 Report Generation

2.1.1 Software

All statistical analyses will be performed using SAS version 9.4 (SAS Institute Inc., Cary, NC, USA) under Microsoft Windows operating system

2.2 Analysis Populations

2.2.1 Completion

Subjects will be considered to have completed the study on the study end date on 8/5/21.

2.2.2 Missing data

We will use data obtained from patient's electronic health record (EHR) to calculate our primary and secondary outcomes. Missing outcome data could occur for several reasons, such as errors of medical documentation or the patient no longer in the primary care registry.

2.3 Statistical Report Contents

The following section outlines the contents of the final statistical analysis. For all analyses, subjects who are enrolled in the study and are eligible will be analyzed in the groups to which they were allocated.

2.3.1 Description of study population

Sample size:
The total number of subjects who are enrolled will be given.

Demographics and Clinical Characteristics, Patient and Parent:
Demographic and clinical characteristics as measured at baseline will be summarized by trial arm. Variables to be summarized are: child age, child sex, child race, child ethnicity, insurance, parent communication preference absence of past patient receipt of measles, mumps, rubella (MMR) vaccine or diphtheria, tetanus and acellular pertussis (DTaP) vaccine as a proxy for childhood vaccine refusal, and patient lifetime historical institutional no-show rate. Variables will be summarized using descriptive statistics appropriate for each type of data item.

Baseline characteristics with large differences between groups will be included in analyses as covariates.

2.3.2 Primary outcome analysis

Primary outcome: Well child care visit completed within 8 weeks of first message sent or date of randomization for the control group.

- Appointment completed (yes or no) based on electronic health record documentation

For the primary outcome, we will use a logistic regression model to examine appointment completion within 8 weeks among the three randomized groups. We converted the odds ratios estimated into risk ratios to ease interpretation. If participants differ in potential covariates across treatment groups, we will conduct additional analyses adjusted for these variables.

2.3.3 Secondary outcome analyses

- Well child care visit scheduled within 2 weeks of first messages sent or date of randomization for the control group.

We will use a logistic regression model to examine appointment scheduling within 2 weeks among the three randomized groups. We converted the odds ratios estimated into risk ratios to ease interpretation. If participants differ in potential covariates across treatment groups, we will conduct additional analyses adjusted for these variables.

- Receipt of 1st dose of COVID-19 vaccination among eligible patients within 8 weeks of first message sent or date of randomization for the control group

We will use a logistic regression model to examine receipt of 1st dose of COVID-19 vaccination within 8 weeks among the three randomized groups. We converted the odds ratios estimated into risk ratios to ease interpretation. If participants differ in potential covariates across treatment groups, we will conduct additional analyses adjusted for these variables.
