# Supplementary material for: Effect of Electronic Outreach Using Patient Portal Messages on Well Child Care Visit Completion: A Randomized Clinical Trial
Source: JAMA Netw Open. 2022 Nov 18;5(11):e2242853. doi: 10.1001/jamanetworkopen.2022.42853 (PMC9675005; doi:10.1001/jamanetworkopen.2022.42853)
Supplement: Supplement 2. — eTable 1. Unadjusted Risk Ratios (RR) for Intent-to-Treat and Per Protocol Analyses eTable 2. Example of Patient Portal Outreach Messages [file jamanetwopen-e2242853-s002.pdf]

Supplementary Online Content

Berset AE, Burkhardt MC, Xu Y, Mescher A, Brinkman WB. Effect of electronic outreach using patient portal messages on well child care visit completion: a randomized clinical trial. *JAMA Netw Open*. 2022;5(11):e2242853. doi:10.1001/jamanetworkopen.2022.42853

eTable 1. Unadjusted Risk Ratios (RR) for Intent-to-Treat and Per Protocol Analyses

eTable 2. Example of Patient Portal Outreach Messages

This supplementary material has been provided by the authors to give readers additional information about their work.

© 2022 Berset AE et al. *JAMA Network Open.*

eTable 1. Unadjusted Risk Ratios (RR) for Intent-to-Treat and Per -Protocol Analyses

Supplemental eTable. Unadjusted Risk Ratios (RR) for Intent-to-Treat and Per-Protocol Analyses

**Outcome**	**Comparison**	**Unadjusted RR (95% CI)**	
		**ITT**	**Per-Protocol**
	**Standard vs. Control**	1.97 (1.32 - 2.83)	1.98 (1.33 - 2.86)
WCC scheduled within 2 weeks	**Tailored vs. Control**	1.57 (1.02 - 2.33)	1.61 (1.05 - 2.41)
	Standard vs. Tailored	1.26 (0.88 - 1.74)	1.23 (0.86 - 1.71)
	**Standard vs. Control**	1.93 (1.38 - 2.60)	1.91 (1.36 - 2.58)
WCC completed within 8 weeks	**Tailored vs. Control**	1.53 (1.06 - 2.13)	1.48 (1.02 - 2.09)
	Standard vs. Tailored	1.26 (0.94 - 1.66)	1.29 (0.94 - 1.71)
	**Standard vs. Control**	4.52 (1.36 – 14.44)	4.97 (1.49 - 15.33)
Receipt of COVID vaccination within 8 weeks	Tailored vs. Control	1.32 (0.22 - 7.18)	0.74 (0.06 - 5.60)
	**Standard vs. Tailored**	3.44 (1.15 - 9.64)	6.25 (1.57 - 24.28)
	Standard vs. Control	0.75 (0.22 - 1.89)	0.74 (0.21 - 1.90)
Receipt of Tdap within 8 weeks [a]	Tailored vs. Control	0.71 (0.20 - 1.81)	0.61 (0.16 - 1.70)
	Standard vs. Tailored	1.06 (0.29 - 2.81)	0.92 (0.25 - 2.50)
	Standard vs. Control	1.88 (0.75 - 3.87)	1.73 (0.66 - 3.71)
Receipt of HPV within 8 weeks [a]	Tailored vs. Control	1.23 (0.42 – 3.00)	0.79 (0.21 - 2.34)
	Standard vs. Tailored	1.53 (0.64 – 2.99)	1.95 (0.73 - 4.23)
	Standard vs. Control	1.01 (0.38 - 2.25)	1.00 (0.37 - 2.24)
Receipt of MCV4 within 8 weeks [a]	Tailored vs. Control	1.15 (0.46 - 2.42)	0.94 (0.34 - 2.15)
	Standard vs. Tailored	0.88 (0.35 - 1.86)	0.88 (0.34 - 1.90)

* The intent-to-treat and per-protocol analyses were adjusted for insurance type, as those in the control group appeared to have less patients with public insurance compared with the Standard and Tailored message groups.

[a] Analysis includes all patients eligible to receive this vaccine during the 8-week study period.

© 2022 Berset AE et al. *JAMA Network Open.*

eTable 2. Example of Patient Portal Outreach Messages

	Monday Message	Thursday Message
**Standard Message**	[Patient first name] is due for a checkup at [clinic name]. We want to keep [patient first name] current so we can address any concerns, complete any needed forms, or help with other needs you may have. Please schedule through [patient portal] or call [clinic phone number] option 1.	[Patient first name] is due for a checkup at [clinic name]. We want to keep [patient first name] current so we can address any concerns, complete any needed forms, or help with other needs you may have. Please schedule through [patient portal] or call [clinic phone number] option 1.
**Tailored Message**	[Patient first name] was last seen for a checkup at [clinic name] on [date of last WCC] and is due for a [patient's current age] checkup. We want to keep [patient first name] current so we can address any concerns, complete any needed forms, or help with other needs you may have. Please schedule through [patient portal] or call [clinic phone number] option 1 to schedule.	[Patient first name] was last seen for a checkup at [clinic name] on [date of last WCC] and is due for a [patient's current age] checkup. We want to keep [patient first name] current so we can address any concerns, complete any needed forms, or help with other needs you may have. Please schedule through [patient portal] or call [clinic phone number] option 1 to schedule.

© 2022 Berset AE et al. *JAMA Network Open.*
