# Supplementary material for: Effect of Electronic Outreach Using Patient Portal Messages on Well Child Care Visit Completion: A Randomized Clinical Trial
Source: JAMA Netw Open. 2022 Nov 18;5(11):e2242853. doi: 10.1001/jamanetworkopen.2022.42853 (PMC9675005; doi:10.1001/jamanetworkopen.2022.42853)
Supplement: Supplement 3. — Data Sharing Statement [file jamanetwopen-e2242853-s003.pdf]

Data Sharing Statement

Berset. Effect of Electronic Outreach Using Patient Portal Messages on Well Child Care Visit Completion. *JAMA Netw Open*. Published November 18, 2022.
doi:10.1001/jamanetworkopen.2022.42853

Data
Data available: No
